# Challenges to access and provision of palliative care for people who are homeless: a systematic review of qualitative research

**DOI:** 10.1186/s12904-016-0168-6

**Published:** 2016-12-03

**Authors:** Briony F. Hudson, Kate Flemming, Caroline Shulman, Bridget Candy

**Affiliations:** 1 Marie Curie Palliative Care Research Department, Division of Psychiatry, UCL, London, UK; 2 Pathway, London, UK; 3 Department of Health Sciences, The University of York, York, UK; 4 Kings Health Partnership, London, UK

**Keywords:** Homelessness, Palliative care, Inclusion health, End of life care, Qualitative, Review

## Abstract

**Background:**

People who are homeless or vulnerably housed are a marginalized group who often experience high rates of morbidity and die young as a result of complex problems. Access to health care and support can be challenging, with access to palliative care even more so. This review presents a synthesis of published qualitative research exploring from the perspective of homeless people and those working to support them, current challenges to palliative care access and provision, in addition to suggestions for what may improve palliative care for this population.

**Methods:**

Systematic review of qualitative research analysed using thematic synthesis. PsycINFO, Medline, Sociological Abstracts, Social Services Abstracts, Science citations index and CINAHL were searched up to September 2016. Thematic synthesis involved a three-step inductive process to develop a deeper understanding of the challenges to and suggestions for the access and provision of palliative care for homeless people.

**Results:**

Thirteen qualitative articles, reporting nine studies were identified. The challenges to access and provision to palliative care were drawn from the data covering three broad areas, namely “the chaotic lifestyles sometimes associated with being homeless”, “the delivery of palliative care within a hostel for homeless people” and provision within “mainstream health care systems”. Obstacles were related to homeless persons competing day-to-day priorities, their experience of stigma in mainstream settings, the high burden on hostel staff in supporting residents at the end of life and inflexibility in mainstream health care systems. Suggestions for improving access to palliative care include building trust between homeless persons and health professionals, increasing collaboration between and flexibility within services, and providing more training and support for all professionals.

**Conclusions:**

The provision of palliative care can be complicated for all populations, however delivering palliative care for people who are homeless is influenced by a potentially greater and more varied range of factors, on both individual and systemic levels, than providing palliative care for the housed population. Careful consideration and potentially great changes will be needed within health care systems to ensure homeless populations have equitable access to palliative care.

## Background

People who are homeless or vulnerably housed include individuals living on the street, sofa surfing, using temporary accommodation systems or hostels. They are referred to, for ease of reading, as homeless people in this paper and are a disadvantaged group who experience higher and different rates of morbidity and mortality than the housed population [1–3]. Their patterns of health care usage differ. In the UK the number of accident and emergency room visits and hospital admissions are reported to be four times higher for homeless people [4], while primary care services are underused [5]. Challenges to homeless people’s access to general health care are varied and multifaceted. They include mistrust of health care professionals [6, 7], perceived stigma and discrimination [8], competing priorities [7], difficulties registering with GPs (due to a lack of fixed address or photo ID) [4] and making [9] and keeping appointments [8].

The reported mean age at death for homeless people ranges from 34 to 47 years, with age-adjusted death rates up to four times higher than the housed population in North American and European cities [1, 10, 11]. In the US and elsewhere, the prevalence of substance or alcohol dependency, psychosis and personality disorder is substantially higher in the homeless than the housed populations [12]. Furthermore, dual diagnoses (severe mental illness and substance misuse) and tri-morbidities (combinations of physical health, mental health and substance misuse issues) are common [13]. Substance and alcohol misuse contribute to almost a third of deaths in the homeless population in the UK [14]. Chronic progressive illnesses such as cancer, liver or respiratory disease are also experienced earlier by the homeless compared with the housed populations [14].

For homeless persons with a terminal illness, deteriorating health, increasing isolation and poor mobility may make access to health care services, particularly specialist care services harder still. This was evidenced in a North American study which reviewed homeless people’s contacts with health services in their last year of life compared with a sample of men aged 45–64 who were housed [15]. The sample from the general population received a mean of 9.5 ambulatory care visits in their last year of life compared with 3.9 ambulatory visits for homeless people in year before death. Furthermore, almost a third of reviewed homeless people (27%) had no health care contacts of any form, in their final year [15]. These homeless people may have benefited from palliative care, a specialised holistic approach to treatment for people with life threatening, or life limiting illnesses. Palliative care aims to improve quality of life and reduce suffering, by attending to the physical, psychosocial and spiritual needs of a person with a terminal illness [16].

Talking about death and dying and putting plans in place for future deteriorations in health can be challenging in any population. However discussions and planning about future care may be further complicated for homeless people due to lack of stable housing or family connections to support implementation of such a plan, a lack of engagement with medical services and personal concerns about stigma and discrimination that may prevent a plan being implemented [8, 17–19].

Given the complexities of providing health care, and more specifically palliative care for the homeless population [7], qualitative research that explores the experiences and views of homeless people and care providers could be especially useful in understanding how best to care for homeless people with advanced ill health. To date, to our knowledge, qualitative studies exploring the views and experiences of people who are homeless, and those supporting them with regards to palliative care have not been synthesised in a qualitative review. Existing reviews in this area have focused on quantitative research, mostly exploring the effectiveness of end of life planning interventions [17, 20].

By undertaking a qualitative synthesis we aimed to generate a deeper understanding of previous research by considering connections, similarities and differences between the data presented in each study. This enhanced understanding may provide more information than the findings of each study in isolation. This may assist in the development of policies, and in services and practices that could facilitate the delivery of palliative care for this currently underserved population.

### Aims

To provide a deeper understanding of the challenges to and suggestions for palliative care access and delivery for homeless people, by synthesising qualitative studies exploring palliative care from the perspective of homeless people and the professionals supporting them.

## Methods

The review was reported according to ENTREQ guidelines (Enhancing transparency in reporting the synthesis of qualitative research) [21]. Since the review sought to aggregate data from qualitative studies to address specific questions relevant to policy and practice, thematic synthesis methods were selected after consulting guidelines [22, 23]. The review was not registered on PROSPERO, as there are no direct health related outcomes.

### Study eligibility criteria

Original peer-reviewed, English language publications of studies that reported using qualitative research methods to explore views regarding palliative care for homeless people were eligible for inclusion. No date restrictions were applied. In mixed methods studies, if qualitative results could be separated from the quantitative analysis, qualitative results were included. Quantitative research, review articles, conference abstracts, non-peer reviewed research and case studies were not included.

We included studies recruiting “homeless people” (aged 18 or over) and professionals working to support them, either as hostel or outreach workers or health and social care providers. For the purposes of this review we defined homeless individuals as living on the street, using temporary accommodation or hostels. This definition has been used in previous research into homelessness conducted in the UK and elsewhere [8, 24]. The homeless people recruited to these studies did not need to have a life limiting condition, but needed to be able to express their views about access to care at the end of life.

### Data collection

Six citation databases were searched from inception to September 2016 for studies meeting the inclusion criteria; PsycINFO, Medline, Sociological Abstracts, Social Services Abstracts, Science citations index and CINAHL. Search strategies were developed based on previous reviews of the wider literature on homelessness [20, 25, 26] and included the following free text and indexed terms;

Homeless* AND death OR dying OR palliative OR end-of-life.

Reference lists and forward searches of included studies were undertaken to identify any further relevant studies. Authors of included studies were contacted to ask if they were aware of any further relevant research.

**Fig. 1:**
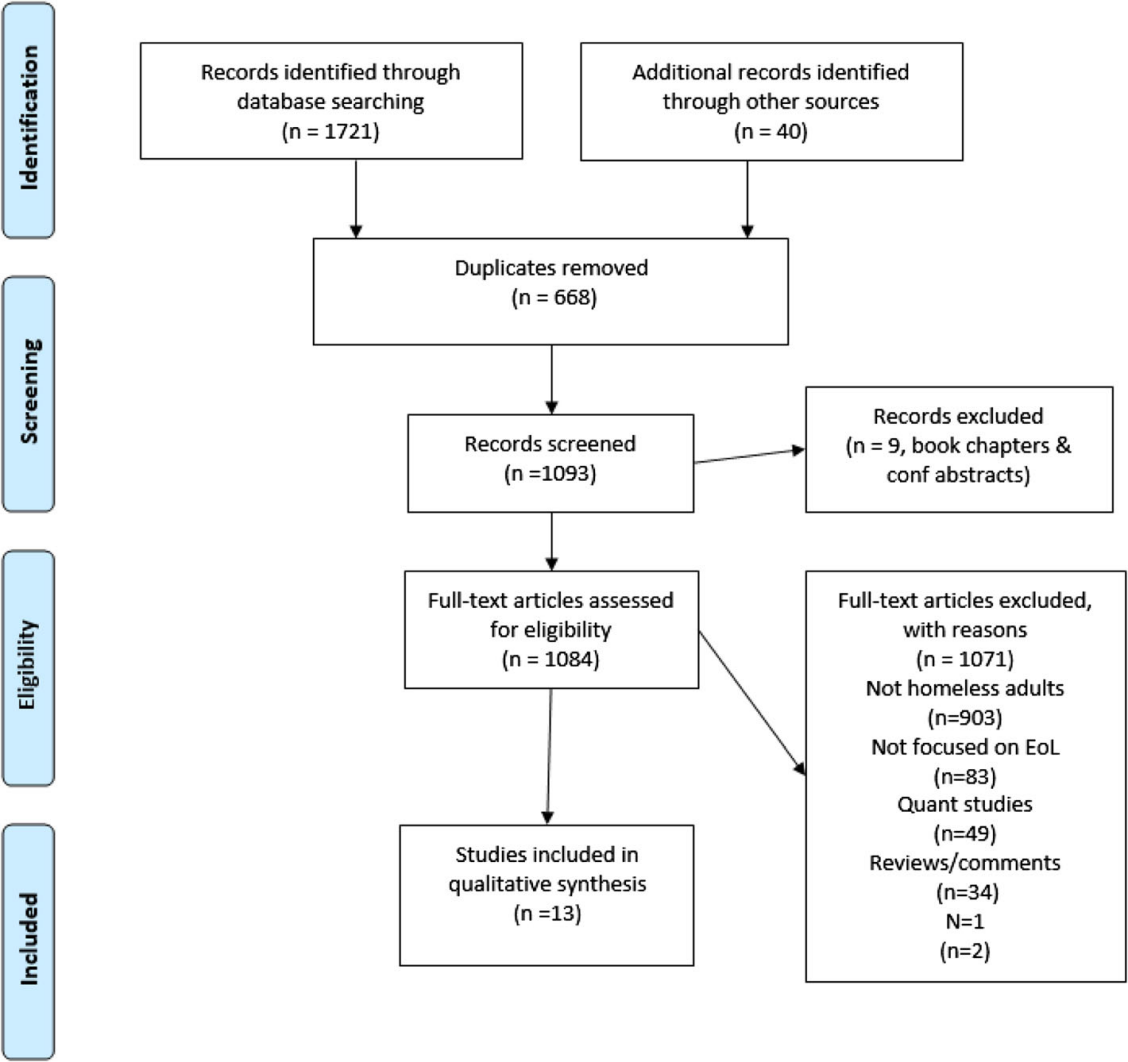
PRISMA flow chart

One reviewer (BH) screened the abstracts of citations generated from electronic searches for eligibility. A second (BC) checked for agreement. Any disputes were resolved through consulting the full text article and discussion. The results of this process are displayed in the PRISMA flow diagram (Fig. 1).

To give an overall impression of the methodological rigor of included studies, a 9-item tool developed by Hawker et al. [27] was used. The items assessed the abstract, reported method, sampling, analysis, ethics and bias, generalisability and implications using the criteria of “good,” “fair,” “poor,” or “very poor” (Table 3 of Appendix 1).

The PDFs of included studies were entered into NVIVO (version 11) for analysis. The following information was extracted; the country in which research was conducted, data-collection method (e.g. focus groups, interviews) the number of participants recruited, participant type (homeless people, health and social care professionals, hostel staff), the analytical approach used and the main findings.

### Data analysis

Previous qualitative syntheses have noted that distinguishing key concepts in the findings of qualitative studies can be difficult, due to inconsistencies in reporting styles and differentiating between data, findings and conclusions [28]. To overcome this only text labelled as “results” or “findings” (both in the abstracts and full texts) were analysed, in order to capture all results without including or being influenced by the conclusions of authors.

Thematic synthesis [15] was used to explore similarities, differences, and relationships between studies and to develop an enhanced interpretation of them [29, 30]. To achieve this, the synthesis involved a three-step inductive process: 1) coding the text, 2) developing descriptive themes and 3) generating analytical themes [28]. These steps did not always follow a strictly sequential pattern and details of each stage are outlined below.

#### Coding the text

BH coded the findings of reviewed studies for salient points relating to palliative care for homeless people. We considered, when organising our coding whether to separate data from homeless people and data from supporting professionals. We found that the data from different types of participants often complimented each other in sentiment therefore we kept data together for readability. The coding was inductive ignoring any predetermined structure. This approach was chosen to account for any context specific detail; for instance what may be a challenge in one situation may be a facilitator in another. Initial codes were reached through discussion and consensus with a second reviewer (BC), who reapplied the codes. Following discussion between reviewers additional codes were developed as necessary. Studies were coded in alphabetical order by first author.

#### Developing descriptive themes

A hierarchy was created among the initial codes through the development of descriptive themes. This served as the first step in “going beyond” the data presented in the studies while remaining grounded in it [28, 31]. Descriptive themes were drafted by BH and reviewed by BC. Consensus was achieved through discussion to ensure that the themes were supported by the original data. These descriptive themes formed the framework for the final analysis on access to and provision of palliative care for homeless people. They were grouped into three broad areas: (1) the chaotic lifestyles sometimes associated with homelessness, (2) providing palliative care within a hostel for the homeless and (3) providing palliative care within mainstream health care systems.

#### Generating analytical themes

The final stage of the analysis involved considering the descriptive themes in relation to the framework of challenges to and suggestions for the improvement of palliative care for homeless people. Descriptive themes, derived from the data were discussed on several occasions indepth by reviewers (BH and BC). Care was taken to ensure that the analytical themes remained true to the dataset but also developed an enhanced understanding of the barriers to palliative care, from the perspective of both the homeless and supporting professionals. Overarching suggestions (generated from the synthesis of the data) for the improvement of palliative care access for homeless people were also developed.

In terms of reflexivity, the authors come from a range of backgrounds, psychology, medicine, nursing and mixed methods systematic reviewing. All researchers have experience in qualitative data collection and analysis, and varying levels of experience in working with homeless people in medical contexts. After synthesis we also consulted a wider group of health care professionals, researchers and an individual with lived experience of homelessness for their opinions on identified themes.

## Findings

### Study characteristics

Figure 1 presents a flow diagram of the study selection process. Our initial search yielded 1721 citations; from this 13 qualitative research papers met our inclusion criteria [6, 9, 32–41, 42], reporting results from 9 studies. No additional studies were found through contacting authors of eligible studies. The study by Neil et al. [36–38] was reported in more than one paper focusing on different aspects of the data, as was the study by Ko et al. [32, 33] and also by Song et al. [39, 40]. No studies were excluded following quality assessment because none were found to be of very poor standard (Table 3 of Appendix 1).

Table 1 outlines the sample, methodology and main results of reviewed studies. Most studies were conducted in the USA or Canada (*n* = 6) with the remaining in Sweden, UK and Australia. Despite a lack of date parameter, no eligible studies published before 2005 were identified. As outlined in Table 1 semi-structured interviews [9, 32–38, 41] and focus groups [39, 40, 42] were the most common methods of data collection.

**Table 1:** Characteristics of papers included in review

Reference	Author	Title	Setting	N	Participants	Data collection methods	Analytical approach	Main findings	Critical appraisal score
[39]	Tarzian, Neal & O’Neil (2005)	Attitudes, Experiences, and Beliefs Affecting End-of-Life Decision-Making Among Homeless Individuals	USA	20	Homeless people	Focus groups	Thematic analysis	Five main themes: 1) Valuing an individual’s wishes; 2) Acknowledging emotions 3) The primacy of religious beliefs and spiritual experience; 4) Seeking relationship-centred care 5) Reframing advance care planning.	27/28
[36]	Song et al. (2007)	Dying on the streets: homeless persons’ concerns and desires about EoLC	USA	53	Homeless people	Focus groups	“Consensual qualitative research” 3 step inductive analytic process	Personal themes: 1) Experience of EoLC 2) Fears and uncertainties around lack of dignity and dying anonymously 3) Preferences wishes and 4) Advance care planning 5) Spirituality & religion 6) Veteran status Relational themes: 1) Relationships with known people/burden to others 2) Relationships with strangers 3) Communication tools Environmental factors 1) Barriers and facilitators to good EoLC 2) Participant suggested interventions	32/34
[37]	Song et al. (2007)	Experiences with and attitudes toward death and dying among homeless persons	USA	53	Homeless people	Focus groups	“consensual qualitative research” 3 step inductive analytic process	Personal themes: 1) Early loss 2) Experience with death 3) Personal life threatening experiences 4) Fears and uncertainties 5) Coping strategies 6) Approach to risk, risk management and risky behaviours Relational themes: 1) Relationships with strangers	33
[29]	Ko, Kwak & Nelson-Becker (2015)	What constitutes a good and bad death?: perspectives of homeless older adults	USA	19	Homeless people (aged 60+)	Semi structured individual interviews	Grounded theory	A good death 1) Dying peacefully 2) Not suffering 3) Experiencing spiritual connection 4) Making amends with significant others. A bad death 1) Experiencing death by accident or violence 2) Prolonging life with life supports 3) Becoming dependent while entering a dying trajectory 4) Dying alone	28/29
[30]	Ko & Nelson-Becker (2014)	Does end-of-life decision making matter? Perspectives of older homeless adults	USA	21	Homeless people (aged 60+)	Semi structured individual interviews	Grounded theory	1) EOL topic is uncomfortable 2) God plans EoLC 3) Physicians are preferred as decision makers 4) EoLC is not a priority 5) need for sensitivity	28/30
[39]	Davis – Berhman (2016)	Serious Illness and End-of-Life Care in the Homeless: Examining a Service System and a Call for Action for Social Work	USA	14	Homeless people, hostel staff, outreach staff and health and social care professionals	Interviews	Grounded theory	Lack of services for serious, chronic and life threatening illness, Barriers to access to services Stigma, End-of-life care.	24
[6]	Hakanson et al. (2015)	Providing palliative care in a Swedish support home for people who are homeless	Sweden	12	Hostel staff	Group and individual discussions	Interpretive description	1) Conditional factors framing palliative care 2) Building trustful- family like relationships 3) Re-dignifying the person 4) Re-defining flexible and pragmatic care solutions	33/34
[38]	Webb (2015)	When dying at home is not an option: Exploration of hostel staff views on palliative care for homeless people	UK	7	Hostel staff	Semi structured individual interviews	Four stage phenomenological method	1) Understanding of palliative care 2) Working with limited medical information 3) Taking responsibility 4) Building rapport 5) Upholding residents dignity 6) Recognising physical deterioration 7) Managing environmental challenges 8) Role limitations and support needs of hostel staff	26/28
[33]	McNeil & Guirguis-Younger (2011)	Illicit drug use as a challenge to the delivery of end-of-life care services to homeless persons who use illicit drugs: Perceptions of health and social care professionals	Canada	50	Canadian Health care professionals and hostel workers	Semi structured individual interviews	Grounded theory	Barriers to EoLC services: 1) Competing priorities 2) Lack of trust of healthcare providers 3) Exclusion from traditional end-of-life care settings Challenges to EoLC service delivery: 1) Non-disclosure of illicit drug use 2) Pain and symptom management 3) Interruptions in care as a result of illicit drug use policies 4) Lack of understanding of addictions and palliative medicine	
[34]	McNeil, Guirguis Younger & Dilley (2012)	Recommendations for improving the end-of-life care system for homeless populations: A qualitative study of the views of Canadian health and social services professionals	Canada	54	Canadian health and social care professionals	Semi structured individual interviews	Grounded theory	Perceived barriers to the EoLC system: 1) Availability of end-of-life services and supports 2) operating policies that exclude homeless populations 3) lack of continuity of care Participant recommendations to improve the EoLC system. 1) Low threshold strategies 2) Partnering community agencies with EoL services 3) Strengthening training for EoLC professionals	31
[35]	McNeil et al. (2012)	Harm reduction services as a point-of-entry to and source of end-of-life care and support for homeless and marginally housed persons who use alcohol and/or illicit drugs: a qualitative analysis	Canada	54	Canadian health and social care professionals	Semi structured individual interviews	Grounded theory	Harm reduction services as a point of entry to EoL services. 1) Increasing engagement with this population 2) Engaging with clients over time 3) Maintaining relationships with EoLC providers Harm reduction outreach services as a source of EoLC: 1) Providing EoLC for those unable to access services 2) Providing EoL support for clients who wished to die at home Residential harm reduction services as a source of EoLC. 1) Providing culturally competent care. 2) Providing EoLC in a home setting 3) Implications of EoLC for regular services	28/30
[31]	Krakowsky et al. (2012)	Increasing access—A qualitative study of homelessness and palliative care in a major urban center	Canada	7	Registered nurses (*n* = 3) & outreach workers (*n* = 4)	Semi structured individual interviews	Thematic analysis	1) Homeless persons’ access to palliative care compromised due to previous negative experiences of Homeless people with the health care system. 2) Staff training needed 3) Palliative services must respect the individual’s habits, friends, and preferred surroundings. 4) Diversity needed in vehicles used to deliver palliative care.	19/21
[32]	Macwilliams et al. (2014)	Reaching out to Ray: delivering palliative care services to a homeless person in Melbourne, Australia	Australia	6	Managers from hospitals, palliative care services & residential shelters.	Semi structured individual interviews	Thematic analysis	Key concerns from health care providers 1) Late stage presentation and multiple admissions 2) Safe use of drugs 3) Non compliance 4) Staff stress	19/21

### Participants

In total, studies represented the views of 98 homeless people, 38 hostel staff, 14 outreach workers and 103 health and social care professionals.

The four North American studies seeking the views of homeless people recruited participants from social service agencies that provided food, shelter and basic health care [9, 32, 33, 39, 40, 42]. Limited information was provided regarding the demographic and socio-educational characteristics of the homeless participants recruited. Ko et al’s. [32, 33] homeless participants had a mean age of 65 and reported a range of conditions including hypertension, heart conditions, diabetes and mental health problems. Fifty-seven percent described their health as very good, 19% as poor. The majority of participants (71%) in Ko et al’s study had experience of living on the street, the average length of time on the streets being 47.3 months. Homeless participants recruited to Tarzian et al’s study [42] ranged in age from 19 to 63 years and were described as “*substance addicted (actively or recovering)*”. Over a third of the participants (35%) in Song et al’s. study [39, 40] were female. Participants had a mean age of 47, 36% were Native Americans and 40% reported experiencing more than one living situation in the last 6 months.

Hostel staff were recruited from intermediate/long stay hostels in the UK [41], an overnight shelter in north America [9] and a support home for homeless people (with medically trained staff) in Sweden [6].

Health and social care professionals were recruited from a range of services in Canada, North America and Australia and had varying degrees of experience in working with homeless people [9, 34–38].

### Results of data synthesis

The views of both homeless people, and those working to support them are represented in the results and data from all studies fed into each theme. The synthesis results are outlined in Table 2.

**Table 2:** Challenges and suggestions for the provision of palliative and end of life care to people who are homeless

Codes	Challenges to the provision of palliative and end of life care	Suggestions for the provision of palliative and end of life care
Challenges related to the chaotic lifestyles associated with being homeless		
Death in the day to day context of homelessness	Previous negative experiences of death and fear of death	Peer advocates/community services to facilitate attendance & engagement
	Unconventional living arrangements & social isolation	Training for staff around addiction issues and associated complications
Attitudes to health care; substance misuse & competing priorities	Previous negative experiences or perceptions of health care & mistrust of professionals	
	Poor engagement with services	
	Substance and alcohol misuse	
	Complex care needs & competing priorities	
	Trends in accessing health care and poor adherence to treatment	
	Communicating about death, dying and advance care planning	
Challenges to the delivery of end of life care and specialized palliative care within a hostel		
The hostel environment	Limited resources	Advocacy
	Difficulty accessing support and specialist services	Greater collaboration with medical services – MDT discussion
	Limited medical information	Greater in hostel support from medical and social services
Practical and emotional burdens for staff	Limitations of staff roles	Increased training & specialised services
	Emotional burden for staff	
Challenges to the provision of palliative care to homeless persons relating to mainstream health care systems		
Inflexibility of the health care service and limited planning	Inflexible services and systems	Flexibility in care model & locations
	Strict rules and regulations	Harm minimisation strategies
	Lack of specialised services	Linking with community services
	Limited planning, especially at discharge	Training for health care professionals
Health care professionals’attitudes and inexperience in supporting homeless people	Attitudes of professionals	Person centred care
	Emotional & practical burdens	Increased training & specialised services

### The chaotic lifestyles sometimes associated with being homeless

The chaotic lifestyles led by many homeless people and issues sometimes associated with being homeless present a range of obstacles to palliative care. Many of these issues individually may not be unique to this population, but their combination and concurrent presentation may be.

Homeless people described the same desires as others for the end of life; compassion, company from someone (sometimes regardless of whether they were a family members or not) and being in a familiar environment [6].

*“…Bad death is being lonely…no friends around you when you’re passing away. Well, death is never really good but…(laughs)…at least it’d be better with … friends around…you know someone to hold your hand and whatever…” – Homeless person* [32]

Yet meeting these wishes may be more complicated for people who are homeless, who often felt abandoned, alone and uncared for:

*“End of life. What end of life are you talking about? … I’m on the street and nobody cares about me” – Homeless person* [33]

For people with more stable lifestyles, family members may be more able to assist in the provision of the support required as health deteriorates. This is less likely to be possible for homeless people [6, 32–35, 39–41]:

*“It makes a difference when you’re homeless and you’re dying…You’re here by yourself…” Homeless person* [40]

At the same time, for some homeless people a terminal diagnosis was seen as a chance to make amends with family members from whom they had become estranged [6, 32]:

*“I’d tell them how much I love them … tell them if I did … forgive me if I did something wrong … Express my feelings and say I love them.. . I want to die comfortably … surrounded by my family…” – Homeless person* [32]

However, this sentiment was not shared by all homeless people or their families:

*“My living Will says my family will have no say or discussion of what is done. Basically, they don’t know me, so why should they have a say in whether I live or not.”- Homeless person* [39].

*“We got in touch with relatives to say that…it’s close now* [death]. *And the relative just says we should throw him on the rubbish pile” – Hostel staff* [6]

### Death in the day to day context of homelessness

Homelessness was portrayed as “*a life filled with fear”* [40], involving repeated exposures to illicit drugs, violence, cold and hunger [6, 32–37, 43]:

*“I’m looking around, taking account of my surroundings, making sure I don’t get jumped” – Homeless person* [40]

These experiences influenced homeless people’s conceptualisations of themselves, others and also society [6]:

*“I think when you’re homeless and you’re out on the street so long, you’re surrounded by grief and death and a lot of stuff. It makes you cold. It makes you unfeeling towards people.”- Homeless person* [40]

The homeless people interviewed described witnessing deaths that were *“more traumatic and demeaning than deaths that domiciled people may experience”* [40] and many had witnessed the deaths of multiple peers [9, 32]. Perhaps as a result, avoidance of thinking or talking about death and dying was common, which acts as a barrier to the establishment of palliative discussions [6, 33, 35–37, 39, 40, 42].

*“Everybody wants to live you know ….I find if I dwell on it, it gets depressing … I get depressed enough you know” – Homeless person* [33]

Furthermore, the social and living conditions associated with homelessness complicate palliative care for this population, who tend to focus on the daily challenges of life, rather than the future and what that may look like:

*“The people that I’ve talked to that live on the street.....they’re just looking to get their food and stay warm…get a shower…They’re not really considering much beyond that”- Homeless person* [33]

### Attitudes to health care; mistrust and competing priorities

In order to examine challenges to palliative care for homeless people, consideration needs to be given to homeless people’s experiences of health care in general. Their understanding of and attitudes towards healthcare provision and practices were informed through these experiences and those of their peers. These were often, as earlier documented, markedly different to the general population. Often shaped by discrimination, disrespect [6] and disempowerment [42]. As a result, expectations and conceptualisation of health care, including of palliative care, described by homeless people appear different to those perhaps commonly shared by the housed population [6, 33, 34, 36, 40]. This contributes to the difficulty homeless people had in trusting or engaging with health and social services [6, 9, 34–37, 39] as outlined in the quote below:

*“There is a lot of shame….low self-esteem, horrific histories of trauma and abuse, mistrust of caregivers. They’ve lived very independent lives. At the end of life, when their needs increase, it’s distressing to them because they need to trust when they’ve never learned to trust. They bring with them experiences that are negative from healthcare providers. It’s a challenge” - Health care professional* [36]

Methods of accessing health care were also influenced by these experiences and this conceptualisation. From the review it was clear that homeless people often generally avoided health care facilities, and crisis presentations of complex symptoms directly to emergency services were common [35]:

*“People who are living on the street…it’s much harder to access them. They don’t come to us and they don’t go anywhere for help until they’re so sick that they’re picked up by an ambulance” – Health care professional*

These patterns of health care usage challenged the implementation of traditional models and methods of palliative care delivery [9]:

*“Those folks die younger and actually die suddenly. The population that I serve often doesn’t make use of [palliative care] facilities. Unfortunately, they die because they have had such poor access they drop dead at the age of 40. I’ve lost two people in their early 50s to sudden death”* – Health care professional [36].

Other factors that influenced how homeless people engaged with health services were related to those who misuse substance and/or alcohol. This may influence how motivated they are in attending appointments, adhering to treatment and how they prioritise their own health [6, 36]. The following quote from a homeless person demonstrates this issue:

*“If you are worried about where your next fix is going to come from or where your next meal is going to come from and you don’t know where you are going to sleep that night, healthcare falls to the bottom of the list” – Health care professional* [36]

Another challenge comes from the non-disclosure of illicit drug use to medical professionals when homeless patients are admitted to hospital. This may result in *“interruptions to care, risk of injury and risk of accidental overdose”* [36]*.* For those that do not wish to stop using substances, zero tolerance drug policies, inflexibility and gatekeeping render many mainstream facilities inaccessible [9, 36, 37]:

*“The people who are addicted to drugs…. we don’t know what they’re going to be like. We ask them not to come back until they’re straight.” – Health care professional* [36]

It is also important to note some differences in how homeless and housed people access health care may also relate in some countries to lack of personal funds and support from the government [9]. However in general, the model of health care related beliefs and behaviour described by homeless participants across studies hinders (irrespective of health care organisation) the delivery of timely, multidisciplinary palliative care.

### Challenges to the delivery of palliative care within a hostel

Hostels for the homeless were not designed to be a place of care for individuals with ill health [38]. As a result, the suitability and appropriateness of people with advanced ill health remaining in hostels has been questioned by some:

*‘The resources thing is—are we really an appropriate environment? Do we have rooms that are equipped for people who are reaching the end of their life?” - Hostel staff* [41]

However, for a number of reasons many homeless people expressed a wish to remain in the hostel should their health deteriorate, rather than be transferred to a hospital [6]. This includes familiarity of the environment, the relationships that may develop between hostel residents and staff, mistrust of health care professionals, dislike of hospital environments or a higher level of comfort and acceptance in the hostel setting. In response, hostel staff tried hard to support the wishes of homeless residents, as far as they could

*“We’ll try to do anything to keep them here because they are family. It’s like they want to die in their home” – Nurse attached to hostel* [38]

*“Certainly, we wouldn’t be able to provide the same level of care that they might receive in the hospital but we might still be more desirable—passing away at home because the program environment had indeed become their home and their community” – Hostel staff* [38]

The resources that hostels do have must obviously be distributed between all residents [36] meaning that providing the support required by a resident with advanced illness may not be sustainable [41]:

*“It got to the point that he had problems going to the bathroom…[hostel] Staff had to basically spend twenty-four hours with this individual. That is when we realized we had nine other residents. Staff were saying we really want to support this client but it’s impossible…. At that point, we said, ‘Okay, we really need to make a referral”- Harm reduction specialist* [38]

The limited specialist resources of hostels greatly reduce the space for choice in place of care and death for homeless people, with hostel staff struggling with the increasing needs of patients with advanced illness:

*“We kind of got caught off guard because our first client that got sick was quite young and experienced profound liver failure, extremely fast. He had to go to the hospital. He didn’t want to but he had to. We didn’t have any nursing support in place. We didn’t have equipment. We didn’t have the drugs. He had to go to the hospital to die” – Hostel staff* [38]

The provision of adequate pain relief in a hostel setting is difficult. Being unable to store or administer medication (particularly opioids) safely in a hostel environment may in itself necessitate transfer to hospital [35], which a homeless person may prefer to avoid [9].:

Hostel staff also may not have accurate or adequate information about residents [6, 9, 41] making the acquisition of the necessary support harder still [6, 35]. This could be because of homeless people’s reluctance to share this information [6, 41] or inadequate information sharing between medical services and hostels [9]. A relationship needed to be established between hostel staff and residents before medical information was shared between them:

*“You have to build a relationship with these people before they will say “I’ve got leukaemia and I’ve got 6 months left.” – Hostel staff* [41]

### Practical and emotional burdens for hostel staff

Hostel staff described “*personal feelings of guilt, trauma, sadness, upset, worry, devastation and stress”* [41] in relation to trying to support a homeless people with advanced ill health. Most hostel staff had minimal, if any, health care training.

*“At the end of the day I’m not a personal carer. My job is not personal care” – Hostel staff* [41]

However, there was a lack of other options [9, 38, 41],

*“It was really hard trying to get someone on the side with us … It was so negative the responses [from mainstream health services] we were getting.” – Hostel staff* [41].

It was clear that hostel staff worked hard to support homeless individuals [6, 35, 38, 39, 41], providing physical and emotional support in addition to advocating to promote quality of life, choice and dignity for homeless people [6, 41]. This may include even supporting them in their free time and beyond the hostel:

*“That weekend when I spent 17 hours at the hospital it was in my own time”- Hostel staff* [11]

Meeting the excessive demands placed upon staff in supporting dying people generates a large strain on hostel staff, which may challenge the provision of compassionate palliative care, as one member of hostel staff described:

*“I think in this line of work, you have to be very resilient … some of the things you are going to come across… and some of the stuff you deal with is going to take you to some very dark places” – Hostel staff* [41]

Both hostel staff [6] and homeless people (as referenced earlier) [9, 33] expressed reluctance or concerns around talking about death and dying, perhaps reflecting society’s general aversion to talking about end of life issues, indicating a level of discomfort with this topic which may further contribute to the burden perceived by hostel staff.

### Challenges relating to mainstream health care systems

While this was an international review, meaning the structure of health care systems differed in different studies, barriers explored were largely related to certain principles underlying services, and to perhaps the attitudes and experiences of those working within them.

### Inflexibility of health care service and limited planning

The lack of and uptake if available of palliative care options in mainstream healthcare for homeless people seems a result of multiple issues including their complex health needs [6, 35, 36], and substance and alcohol misuse [35, 36], being uncomfortable in institutional settings [34], and thereby general avoidance of medical services and personnel [6, 34, 36], and subsequently late presentation to services [6, 9, 35, 37].

*“The health care system has failed that population…When trying to access care in the mainstream, they experience discrimination and disrespect and poor care”- Health care professional* [37]

Frustration was expressed about the lack of palliative health care services that were available for homeless people [9], particularly those that could provide palliative care in an environment in which homeless people would feel comfortable and which would be accepting of them [6, 9, 34–37, 39, 41, 42]. This lack of options resulted in the deaths of homeless people in unacceptable circumstances, including on the streets:

*“People died outside on the streets because [end-of-life care providers] couldn’t provide that”- Emergency shelter director* [37]

The way homeless people were portrayed in the included studies, gave the impression of a population not used to operating under the constraints of health service regulations [6, 36, 37]. As such, the inflexibility of mainstream services challenged the ability of homeless people to access them. For homeless people misusing substances or alcohol, particular obstacles to palliative care were evident, including communication difficulties and reluctance of staff to admit them because of unpredictable behaviour [6, 35–38]. Substance misuse may also mask symptoms, making illness assessment and prognostication harder still [6, 36]. Futhermore, policies including zero tolerance, which prohibit the use of alcohol and substances rendered mainstream palliative care facilities inaccessible to some of the homeless population [9, 36, 37], as illustrated in the following quote:

*“The clients are still on drugs. They go out, pick up some crack cocaine and they’re using it. In main stream settings, you’re not going to get that. That’s not going to happen. Traditional hospices are very rigid. There’s no flexibility around behaviours. If someone gets angry or says something wrong, they’re asked to leave” – Health care professional* [36]

A lack of continuity and planning by health and social care professionals was also described, particularly around hospital discharge [9, 35, 37]. This may be in response to the inadequacies of existing services and the scarcity of options available to homeless people with advanced care needs:

*“The hospital social workers will many times release the people back here to the shelter that are completely inappropriate to be in a shelter. We are not a nursing care facility.” – Hostel staff* [9]

### Health care professionals’ attitudes and inexperience

As homeless people do not form a large proportion of the patient group the majority of health care professionals treat, many do not have the training or the experience needed to provide appropriate palliative care to homeless people and thereby find it difficult to meet their particular needs.

*“When you’re trained in your profession, you’re trained in a certain way. If harm reduction wasn’t in your training, you’re not going to know anything about it. How can you expect somebody to embrace that with open arms if they know nothing about it?” - Harm Reduction Specialist* [37]

Health professionals described experiencing *“feelings of failure or lack of achievement”* [35] when working with homeless people. Treating an individual with needs that are very different to those of their usual patient group, with little training could limit the provision of quality palliative care for this population [36, 37]. Inexperience in caring for people who are homeless may contribute to some of the attitudes health care professionals may hold towards homeless and may also contribute to their perceptions of stress and burden.

It was clear from this review that homeless people perceived negative attitudes from health care providers [6, 34–37, 39]:

*“I got out* [of the hospital] *and I’m walking, really sick, carrying my bags, and there was nowhere really to go…the doctors made it clear that my life was not their problem…” - Homeless person* [40]

Perhaps in response, a mistrust of health care professionals was reported [6, 34, 38, 42] which challenged the development of relationships between homeless people and services providers, and subsequently, palliative care access.

Moreover, negative stereotypes contributed to the attitudes towards homeless people and mistrust between homeless people and health care providers:

*“A lot of people have the idea that…[homeless people] are drug addicts and have mental health issues. That’s the case for some, but we’re seeing a different face of homeless now. We have patients in the clinic who have doctoral degrees that lost their jobs and they just can’t find work.– Social worker* [9]

### Improving palliative and end of life care for homeless people– suggestions derived from studies

Across the studies, homeless people and those working to support them shared their views as to how access to health care services including palliative care could be improved.

### Building trust and relationships

In facilitating access to palliative care, building or rebuilding trust between homeless people and health care professionals was considered vital by homeless people [33, 35, 42] and those supporting them [6, 9, 37, 38]. It was recognised though that this would not be easy:

*“You have to earn it. You have to show that you want to do something for them [*homeless people]. *You have to be respectful and treat people with the same kind of treatment that you would want. It's often word of mouth. One client will say, “Listen, you can trust her” - Harm reduction outreach worker* [38]

Building trusting relationships, despite initial mistrust and suspicion [6] may help to facilitate choice, compassion and understanding at the end of life [9], which may contribute to more dignified deaths for homeless people. The use of peer mentors to help support homeless people in accessing services [37] was championed in achieving this aim:

*“It would be helpful to have like individuals who serve as bridges between the [health and social services] systems…. I think that people are the key to building bridges” – Health care professional* [37]

### Collaboration between professionals in the care and support of homeless people

Promoting between community and health services partnership, through the utilisation of existing relationships could facilitate the identification of homeless people that may benefit from palliative care services, at an earlier point in their illness [38]. Developing relationships with community services, such as shelters, soup kitchens and syringe exchange programs may help medical services and professionals to make contact with homeless people in environments in which they are more comfortable [9, 36, 41, 42]. Because of their consistent and prolonged contact and relationships with homeless people, professionals working within hostels and in other community settings could act as links between homeless people and health care services:

*“To avoid unnecessary disrespect and bad treatment of the patients, staff also went to great lengths to inform workers in other departments about these matters. For example, staff would talk with the X-ray department when these patients were scheduled to come there” – Hostel staff* [6]

Community based staff may also be in a position to advocate for homeless people in health care situations, due to their longer term relationship and thereby understanding of the individual’s needs;

*“Three or four of these clients since I’ve started working here have been recognized by the workers at [harm reduction program]. They know to call us and that we’ll follow through with helping with appointments and referrals to the [EoLC]” – Health care professional* [38]

### Flexibility within health services

Current mainstream palliative services cannot adequately support homeless people given their “*structure and incompatibility with lifestyles associated with illicit drug use*” [34]. Additional flexibility is needed to ensure homeless people are able to access and benefit from palliative care services [6, 35, 37].

*“We agreed to walk outside on the street with these people. [Harm reduction] is part of walking down the road, so that they don’t go out and drink Listerine” - Emergency shelter director* [37]*.*

This strategy may ease some of the burden on hostel staff, in addition to potentially improving the quality of life for homeless people who wish to remain out of hospital [34].

Professionals need to demonstrate their ability to treat homeless people respectfully, and as individuals [39, 41, 42]. The need to honour people’s wishes was echoed by homeless people in this research:

*“You respect the wishes of the one dying, That’s the main thing” − Homeless person* [42]

Suggestions for overcoming the complex needs and irregular lifestyles of homeless people in the delivery of palliative care included taking a pragmatic, person-centered approach [6, 34], setting goals that are realistic in the context of homelessness [6] and removing discrimination and stigma from health care interactions [33, 36, 37, 42].

### Training and support for professionals working with homeless people

The complexities of homelessness and issues associated with homelessness were not always understood by health and social care professionals [6, 35–40, 44]. As a result, developing the relationships needed for palliative care discussions to take place was described as difficult [34–37, 39, 40].

*“The unwelcomeness from the medical staff is a big issue. That’s the major one that really needs to be addressed and I feel…there needs to be a lot of education… to overcome this barrier. I understand there are issues of hygiene and behavioural problems but I think … we could tear down a lot of these barriers” - Social worker* [37]

Many studies called for more training for health and social care professions and exposure to the realities of homelessness to promote insight and understanding into the lives and experiences of homeless people [9, 36, 37, 39–41, 44].

*“Have a doctor, an intern, or…a medical student come and work at a shelter for a week, just to see how it is. To get woke up at 6:00 in the morning and booted out… getting a cold bowl of cereal… for breakfast, and just shadowing somebody that… is homeless… if just to say ‘I know this guy; he’s homeless and this needs to be taken care of right away… not making him wait. Then they will have an ideal of what it’s like being homeless” - Homeless person* [40]*.*

## Discussion

### Summary of findings

The provision of palliative care to homeless people is complicated and challenging. This review aimed to explore the challenges to and suggestions for the improvement of access to palliative care, from the perspectives of homeless people and those supporting them.

The barriers to palliative care drawn from the data cover three broad areas (1) the chaotic lifestyles sometimes associated with being homeless, (2) the delivery of palliative care within hostels for the homeless and (3) the delivery of care within mainstream health care systems. Much work is needed to promote trust between homeless people and the services that serve them, and collaboration between services to promote an integrated approach to care. Health care systems need to incorporate a greater degree of understanding and flexibility in order to be accessible to the homeless population and staff may require greater support and training in order to manage the emotional and practical burdens associated with their work.

### Comparisons to other literature and the wider context

The findings of this review are in line with previous research, which has concluded the need for greater flexibility, communication and collaboration within and between services in order for them to be accessible to homeless people [6, 45–47]. An example of this in practice comes from the UK charity Pathway that aims to improve the quality of health care provided to homeless people [48]. Pathway have established within hospitals across the UK, both on wards and in emergency departments, dedicated teams of health and social care professionals and experts by experience, who provide in-hospital advocacy and support for homeless people and promote safe discharge interventions. Pathway teams are not focused specifically on palliative care, yet this model has been shown to improve outcomes for patients (expressed through reduced emergency service attendances) and also provide cost savings for hospitals [48–50].

Also in the UK, a resource pack for hostel staff has been developed by Marie Curie and St Mungo’s [51, 52] to help hostel staff identify residents that may benefit from palliative care and to provide information about palliative care and how to support residents. Research is currently underway to refine and extend this training to additional professional groups [53, 54]. Furthermore in hostels for the homeless run by St Mungo’s, a palliative care co-ordinator has been appointed to increase collaboration between services, to advocate for access to these services and to support both hostel residents and staff.

Further examples of flexible, person-centred services for homeless people can be found in Canada. The PEACH (palliative education and care for the homeless) programme, an interdisciplinary team of health and social care professionals that a take medical care outside of the hospital environment and into the community [55]. In addition, a shelter based hospice has also been established in Canada [56] which provides care in an environment which is acceptable to homeless people, is sensitive to their needs and utilises a harm reduction approach. The benefits of this type of service were evident in the expressions of gratitude and appreciation from service users and also in the associated cost savings [56].

The development of trusting relationships between health and social care services and homeless people was identified in this review as a potential mechanism through which to facilitate access to palliative care for homeless people. One suggestion for achieving this was the use of peer mentors, or experts by experience who could accompany, mentor or advocate for homeless people as they try to access health care services. Previous work from Groundswell in the UK has found that the use of peer mentors can be effective in increasing the confidence and motivation of homeless people to access health care and in decreasing reliance on unplanned secondary care services [57]. This may well be a model that could be extended for homeless people with advanced ill health.

### Limitations

There are a number of limitations to this review that warrant consideration. While research in this area is growing, the pool of evidence from which to draw conclusions is mostly from North America. We acknowledge that differences in context, including health care systems as a result of the inclusion of international research, will limit generalisations, however we feel that aspects of the experience of homelessness and access to services identified, cross national borders. The majority of studies reported the results of service providers, however we tried to ensure that the voices of homeless people, where present were fully represented in this paper.

As a result of the complexities of the interactions between homeless people, attitudes to health, health services and palliative care, and as with all qualitative research, our synthesis may not represent the only interpretation of the data reviewed. Through the extensive analysis process and after much discussion between reviewers, we feel that our analysis represents an enhanced understanding of the reviewed data. Additionally, opinions of experts (an individual with lived experience of homelessness, a range of medical professionals who specialise in health care, including palliative care for the homeless population and the medical director of a charity aiming to improve homeless health in the UK) in this area were sought to confirm this. It is important to highlight that we selected thematic synthesis because of its capacity to help answer several questions (in this review the challenges and suggestions for overcoming them), it is also an approach recommended for use when the answers sought are of relevance to policy and practice [23]. The data we used was also limited in contextual depth, such as service provision and characteristics of homeless persons, thereby restricting our choice of synthesis method.

### Implications for future research

Further research, in particular research outside of North America that provides a platform for the voices of homeless people around the complexities of palliative care for this population is imperative. This may not be easy to achieve as an aversion to talking about death and dying among homeless people and staff was identified in this review (6, 3 (9) 0–32, 35, 39, 40 (9)). Furthermore, difficulties in recruiting homeless participants for research have been reported in previous studies [38]. Of the included studies, half the studies that recruited homeless people provided vouchers as an incentive for participation. Strategies and considerations for effective research recruitment in this population would benefit from exploration.

Future research should ensure, where possible, information about homeless participants’ current and past living arrangements and health status is included. This may enable firmer conclusions to be drawn about factors that influence palliative care access. For example, comparisons between the challenges faced by rough sleepers and hostel residents may delineate the role that hostels and hostel staff play in promoting access to palliative care services and supports.

## Conclusions

The provision of palliative care can be complicated for all populations, however delivering palliative care for people who are homeless is influenced by a potentially greater and more varied range of factors, on both individual and systemic levels, than providing palliative care for the housed population. Much consideration and potentially great changes will be needed within health care systems to ensure homeless populations, and the people working to support them have equitable access to palliative care.

## Appendix 1

**Table 3:** Quality appraisal scores (using tool from Hawker et al. 2002 [27])

Source Paper (*n* = 12)	Title of paper	Abstract/Title	Intro/Aims	Method/Data	Sampling	Data Analysis	Ethics/Bias	Results	Transferability	Implications	Comments	Quality score (out of 36)
Hakanson et al., (2015) [6]	Providing Palliative Care in a Swedish Support Home for People Who Are Homeless	4	3 – literature review good but no objectives	4 – good description of data collection and recording methods	2/3 – no justification of sampling size but reports that all staff at the hostel were invited…?	4	4 – good description of ethical considerations. Researchers role reflected upon in design section	4 – finding relate to aims and are supported by quotes	4 – the context and setting of the research are well described	4 – insights from Sweden, ideas for research – perspectives of participants, practice – apply interventions based on this research to other pall care settings for HP		33/34
Ko & Nelson-Becker (2014) [33]	Does end-of-life decision making matter? Perspectives of older homeless adults	4	2/3 – no objectives. Literature review brief	3 – brief but includes some of the Qs asked.	2/3 recruitment no justification of sample size.	4	2 – insufficient detail for full assessment to be made	4 – clear & relate to aims, supported by quotes	3 – setting more needed to replicate	4 – insights of older HP, research – mixed site, practice – recommendations for discussing EoL		28/30
Ko, Kwak & Nelson-Becker (2015) [32]	What Constitutes a Good and Bad Death?: Perspectives of Homeless Older Adults	4	3 – no objectives, good lit review	3 – Brief but includes some of the Qs asked.	2/3 recruitment & participant characteristics described, no justification of sample size.	4	2 – insufficient detail for full assessment to be made	4	2 setting- more needed to replicate	4 – themes relating to good death for older HP – research – include HP from multiple sites – practice – HCP need better understanding of HP and EoL	Same participants as previous study	28/29

## Abbreviations

EoLC: End of life care
HP: homeless people
